# Supplementary material for: Risk of Avascular Necrosis with The Modified Dunn Procedure in SCFE Patients: A Meta-Analysis
Source: Children (Basel). 2022 Oct 31;9(11):1680. doi: 10.3390/children9111680 (PMC9688411; doi:10.3390/children9111680)
Supplement: Supplementary file 1 [file children-09-01680-s001.zip › children-1977471-supplementary.pdf]

Supplementary Materials

Table S1. Study Details.

Author, Year	Number of patients	Number of cases	N Stable Loder	N Unstable Loder	Mean age in years (range)	Mean follow-up in months (range)
Galletta et al., 2021 [28]	76	81	81	0	13.6 (9 to 17)	68.4 (40 to 144)
Agashe et al., 2020 [29]	30	30	19	11	13.05 (11 to 15.5)	25.36 (13 to 60)
Passaplan et al., 2020 [30]	17	18	14	4	12.9 (6.8 to 17)	113 (50 to 250)
Zuo et al., 2020 [31]	20	21	20	1	13.2 (10 to 17)	31.2 (12 to 57)
Masquijo et al., 2019 [32]	20	21	6	15	12 (10 to 16)	40.4 (12 to 84)
Novais et al., 2019 [33]	27	27	0	27	12.6 (11.5 to 13.8)	30 (22 to 37)
Ebert et al., 2019 [34]	15	15	15	0	12.9 (11.8 to 15)	46 (12 to 120)
Davis et al., 2019 [35]	44	48	17	31	12.94 (NR)	32.9 (NR)
Sikora-Klak et al., 2019 [36]	14	14	14	0	13.1 (NR)	29 (18 to 40)
Jackson et al., 2018 [37]	9	9	0	9	14 (9 to 15)	22 (NR)
Trisolino et al., 2018 [38]	15	15	15	0	13.9 (NR)	44.4 (24 to 64)
Ziebarth et al., 2017 [39]	43	43	38	5	13 (9 to 18)	144 (120 to 204)
Abdelazeem et al., 2016 [24]	31	32	32	0	14.3 (10.5 to 17)	24.1(12 to 40)
Novais et al., 2015 [40]	27	27	0	27	13 (8 to 17)	12 (12 to 96)
Novais et al., 2015 [41]	15	15	15	0	14 (12 to 17)	30 (12 to 72)
Souder et al., 2014 [42]	17	17	10	7	12.2 (9.3 to 16.7)	15.6 (8 to 33)
Upasani et al., 2014 [43]	43	43	17	26	12 (9 to 17)	31.2 (12 to 96)
Arora et al., 2013 [44]	8	8	0	8	13.1 (10 to 16)	20.15 (4 to 48)
Madan et al., 2013 [25]	28	28	11	17	12.9 (10 to 20)	38.6 (24 to 84)
Sankar et al., 2013 [22]	27	27	0	27	12.5 (9.7 to 16)	22.3 (12 to 48)
Alves et al., 2012 [45]	6	6	0	6	12.5 (11 to 15)	20.16 (12 to 26)

Masse et al., 2012 [20]	20	20	18	2	14.3 (9 to 19	24 (13 to 42)
Huber et al., 2011 [21]	28	30	25	3	12.2 (9.4 to 16.6)	45.6 (12 to 102)
Slongo et al., 2010 [46]	23	23	3	20	11.9 (7 to 17)	29.3 (23 to 62)
Ziebarth et al., 2009 [47]	40	40	27	12	12.8 (9 to 18)	54.8 (12 to 101)
Leunig et al., 2007 [13]	30	30	NR	NR	13 (10 to 17)	55 (24 to 96)
N, Number; SCFE, Slipped Capital Femoral Epiphysis						

Table S2. Outcome Metrics.

Author, Year	HHS Overall (range or SD)	HHS Stable (range or SD)	HHS Unstable (range or SD)	Other Outcome Metrics Points (range or SD)	Other Outcome Metrics
Galletta et al., 2021	98.8 (78 to 100)	NR	NR	1 (0 to 12)	WOMAC score
Agashe et al., 2020	81.83 (7.1)	NR	NR	NR	NR
Passaplan et al., 2020	88.7 (52 to 100)	93.10	73.20	16.5 (14 to 18)	Merle d'Aubigne
Zuo et al., 2020	96.7 (13.4)	NR	NR	95.4 (10.6)	WOMAC score
Masquijo et al., 2019	76.3 (40 to 100)	NR	NR	NR	NR
Novais et al., 2019	NR	N/A	NR	NR	NR
Ebert et al., 2019	85.7 (12.1)	85.7 (12.1)	N/A	NR	NR
Davis et al., 2019	NR	NR	NR	NR	NR
Sikora-Klak et al., 2019	NR	NR	N/A	NR	NR
Jackson et al., 2018	NR	N/A	NR	NR	NR
Trisolino et al., 2018	NR	NR	N/A	85 (74-97)	Non Arthritic Hip Score
Ziebarth et al., 2017	NR	NR	NR	17 (14 to 18)	Merle d'Aubigne
Abdelazeem et al., 2016	96.3 (13.2)	96.3 (13.2)	N/A	12.1 (11 to 14)	Merle d'Aubigne
Novais et al., 2015	NR	NA	NR	NR	NR
Novais et al., 2015	NR	NR	N/A	NR	NR

Souder et al., 2014	NR	NR	NR	NR	NR
Upasani et al., 2014	NR	NR	NR	NR	NR
Arora et al., 2013	NR	N/A	NR	16.8 (12 to 18)	Merle d'Aubigne
Madan et al., 2013	89.1 (9)	90.30	88.20	N/A	N/A
Sankar et al., 2013	88.7 (16.4)	N/A	96.70	9.3 in non-AVN, 5.9 in AVN	UCLA score
Alves et al., 2012	NR	N/A	NR	NR	N/A
Masse et al., 2012	98.2 (3.2)	98.56	95.00	Pain 0.6 (0 to 4), Function 2.2 (0 to 12)	WOMAC score
Huber et al., 2011	97.8 (8.2)	97.69	98.33	Pain 5.9 (NR), Function 5.7 (NR)	WOMAC score
Slongo et al., 2010	99 (4.04)	NR	NR	17 (1.6)	Merle d'Aubigne
Ziebarth et al., 2009	NR	NR	NR	NR	NR
Leunig et al., 2007	NR	NR	NR	NR	NR
N, Number; SD, Standard Deviation; NR, Not Reported; N/A, Not Applicable; HHS, Harris Hip Score					

Table S3. Revisions in Cases Which did Not Develop AVN.

Author, Year	Revisions in Non-AVN cases	N of Non-AVN SCFE cases	Rate of Revisions in Non-AVN SCFE cases	Specific Details of Revision
Galletta et al., 2021	7	65	10.8%	1 case of HO, 2 cases for fixation failure, 1 with femoral neck non-union, 3 with ROH for hardware intolerance
Agashe et al., 2020	1	28	3.6%	One patient with hip subluxation, requiring open reduction
Passaplan et al., 2020	4	16	25.0%	6 non-AVN related revision surgeries in 4 total patients. One patients required ROH, 1 patient required offset correction followed by ROH, 1 patient required arthroscopic offset correction, 1 required removal of hardware followed by open offset correction
Zuo et al., 2020	1	21	4.8%	One early implant failure leading to conversion to PAO
Masquijo et al., 2019	NR	NR	NR	Does not differentiate between non-AVN and AVN related revisions. 10 total revision procedures in 21 total patients.
Novais et al., 2019	NR	NR	NR	Seven of 27 total patients had unplanned procedures however does not differentiate if these cases of AVN of non-AVN related revisions

Ebert et al., 2019	2	11	18.2%	One patient with LLD requiring correction, one open reduction and loose body removal for hip instability
Davis et al., 2019	3	10	30.0%	Post-operative hip subluxation or dislocation requiring revision
Sikora-Klak et al., 2019	1	10	10.0%	1 case of LLD requiring revision
Jackson et al., 2018	NR	7	NR	None
Trisolino et al., 2018	NR	NR	NR	None
Ziebarth et al., 2017	14	43	32.6%	9 hips underwent screw removal, four hips underwent offset correction, two with open acetabular rim trimming and offset correction
Abdelazeem et al., 2016	1	31	3.2%	one hip developed deep infection with AVN and ultimately required hip fusion, one hip developed completed head collapse and required screw removal
Novais et al., 2015	NR	NR	NR	None
Novais et al., 2015	1	14	7.1%	1 removal of intra-articular penetrating pin, 1 revision of fixation due to implant failure, and then THA for treatment of osteonecrosis
Souder et al., 2014	NR	13	NR	None
Upasani et al., 2014	NR	33	NR	15 revision procedures for femoral head AVN, fixation failure, or postoperative dislocation.
Arora et al., 2013	NR	6	NR	None
Madan et al., 2013	0	24	0.0%%	None
Sankar et al., 2013	3	20	15.0%	4 broken implant revisions, 3 successful. 3 revisions successful, 1 developed AVN due to noncompliance with weight-bearing restrictions.
Alves et al., 2012	2	2	100.0%	One successfully revised for K-wire fixation failure, another successfully revised for limb length discrepancy.
Masse et al., 2012	1	20	5.0%	One successful revision for intra-articular wire penetration.
Huber et al., 2011	4	29	13.8%	4 failed with cortical screw (out of 20 treated with cortical screw)
Slongo et al., 2010	NR	22	NR	None
Ziebarth et al., 2009	3	40	7.5%	Three successful revisions at 6 to 8 weeks post-op for screw or wire fixation breakage.
Leunig et al., 2007	3	30	10.0%	Two cases were screw fixation failures, third case was a bent full threaded wire. All three successfully revised.

N, Number; SCFE, Slipped Capital Femoral Epiphysis; AVN, Avascular Necrosis; NR, Not Reported

Table S4. AVN in SCFE Treated with the Modified Dunn Procedure.

Author, Year	Overall % of AVN (N/cases)	% of Stable w/AVN (N/cases)	% of Unstable w/AVN (N/cases)
Galletta et al., 2021	19.8 % (16/81)	19.8 % (16/81)	N/A
Agashe et al., 2020	6.7% (2/30)	0.0% (0/19)	18.2% (2/11)
Passaplan et al., 2020	11.1% (2/18)	14.3% (2/14)	0.0% (0/4)
Zuo et al., 2020	0.0% (0/21)	0.0% (0/20)	0.0% (0/1)
Masquijo et al., 2019	47.6% (10/21)	33.3% (2/6)	53.3% (8.15)
Novais et al., 2019	25.9% (7/27)	N/A	25.9% (7/27)
Ebert et al., 2019	26.7% (4/15)	26.7% (4/15)	N/A
Davis et al., 2019	14.6% (7/48)	29.4% (5/17)	6.5% (2/31)
Sikora-Klak et al., 2019	29.0% (4/14)	28.6% (4/14)	N/A
Jackson et al., 2018	22.2% (2/9)	N/A	22.2% (2/9)
Trisolino et al., 2018	20.0% (3/15)	20.0% (3/15)	N/A
Ziebarth et al., 2017	0.0% (0/43)	0.0% (0/38)	0.0% (0/5)
Abdelazeem et al., 2016	6.3% (2/32)	6.3% (2/32)	N/A
Novais et al., 2015	25.9% (7/27)	N/A	25.9% (7/27)
Novais et al., 2015	6.7% (1/15)	6/7% (1/15)	N/A
Souder et al., 2014	23.5% (4/17)	20.0% (2/10)	28.6% (2/7)
Upasani et al., 2014	23.3% (10/43)	17.6% (3/17)	26.9% (7/26)
Arora et al., 2013	25.0% (2/8)	N/A	25.0% (2/18)
Madan et al., 2013	14.3% (4/28)	0.0% (0/11)	23.5% (4/17)
Sankar et al., 2013	25.9% (7/27)	N/A	25.9% (7/27)
Alves et al., 2012	66.7% (4/6)	N/A	66.7% (4/6)
Masse et al., 2012	0.0% (0/20)	0.0% (0/18)	0.0% (0/2)
Huber et al., 2011	3.3% (1/30)	4.0% (1/25)	0.0% (0/3)

Slongo et al., 2010	4.3% (1/23)	33.3% (1/3)	0.0% (0/20)
Ziebarth et al., 2009	0.0% (0/40)	0.0% (0/27)	0.0% (0/12)
Leunig et al., 2007	0.0% (0/30)	NR	NR

N, Number; SCFE, Slipped Capital Femoral Epiphysis; AVN, Avascular Necrosis; N/A, Not Applicable; NR, Not Reported
